# Impact of statin use on breast cancer recurrence and mortality before and after diagnosis: a systematic review and meta-analysis

**DOI:** 10.3389/fonc.2023.1256747

**Published:** 2023-12-18

**Authors:** Xiaolin Jia, Ye Lu, Zili Xu, Qingqing Mu

**Affiliations:** ^1^ Department of Infectious Diseases, The Third People’s Hospital of Longgang Shenzhen, Shenzhen, China; ^2^ Department of Breast Surgical Oncology, National Cancer Center/Cancer Hospital, Chinese Academy of Medical Sciences and Peking Union Medical College, Beijing, China; ^3^ Clinical Medicine School of Zhengzhou University, Zhengzhou, China

**Keywords:** statins, breast cancer, overall mortality, disease-induced mortality, recurrence

## Abstract

**Objective:**

Breast cancer is one of the most common causes of death among women. Statins, typically used for cholesterol management, have been hypothesized to reduce recurrence and mortality rates in breast cancer. However, this association remains a subject of debate. This study evaluates the potential impact of statins on breast cancer recurrence and mortality.

**Methods:**

A comprehensive search was conducted in the PubMed, EMBASE, and Cochrane databases for articles published up to June 2023. These articles examined the effect of statins on breast cancer recurrence and mortality both before and after diagnosis. The analysis was performed using random-effects models, calculating pooled hazard ratios (HR) and their 95% confidence intervals (CI).

**Results:**

A total of 31 cohort studies, involving 261,834 female breast cancer patients, were included in this analysis. It was found that statin use prior to diagnosis was associated with a decrease in overall mortality (HR, 0.8; 95% CI, 0.69–0.93; I2 = 77.6%; *P* = 0.001) and breast cancer-specific mortality (HR, 0.76; 95% CI, 0.67–0.87; I2 = 72.7%; *P* = 0.005). Additionally, statin use after diagnosis was observed to reduce the recurrence of breast cancer (HR, 0.71; 95% CI, 0.61–0.82; I2 = 60%; *P* = 0.003), overall mortality (HR, 0.81; 95% CI, 0.70–0.92; I2 = 80.7%; *P* < 0.001), and breast cancer-specific mortality (HR, 0.76; 95% CI, 0.67–0.86; I2 = 74.5%; *P* < 0.001).

**Conclusions:**

The findings of this study indicate that statin usage, both before and after breast cancer diagnosis, may be associated with reduced risks of overall and breast cancer-specific mortality, as well as lower recurrence rates.

## Introduction

Breast cancer remains a major health issue for women worldwide. It is a leading cause of cancer-related deaths (1, 2), representing a quarter of all female cancer fatalities. In America, it ranks second only to lung cancer in terms of mortality rates (3, 4). While developed countries have seen a decrease in breast cancer incidence and improved outcomes, the situation is worsening in developing nations (5) due to limited treatment options. This highlights the urgent need for new and effective treatments.

Statins, primarily prescribed for treating dyslipidemia to reduce the risk of atherosclerosis and cardiovascular events (6, 7), are now increasingly being explored for their potential anti-cancer properties, extending beyond their well-known cardiovascular benefits (8, 9). Initial studies suggest that statins may be effective in a variety of cancers (10), potentially by inhibiting cell proliferation and inducing apoptosis (11, 12). The efficacy of statins appears to depend on their lipophilicity, dosage, and duration of treatment (10). However, due to patient diversity, there are discrepancies between laboratory studies and clinical data. Some studies have indicated a correlation between statin use and reduced breast cancer mortality (13–15), while others have found no such association (16–18). This study, therefore, aims to clarify the relationship between statin use and the risk of mortality or recurrence in women with breast cancer.

## Methods

This meta-analysis adhered to the Preferred Reporting Items for Systematic Reviews and Meta-Analysis (PRISMA) guidelines.

### Search strategy and inclusion criteria

**Figure 1:**
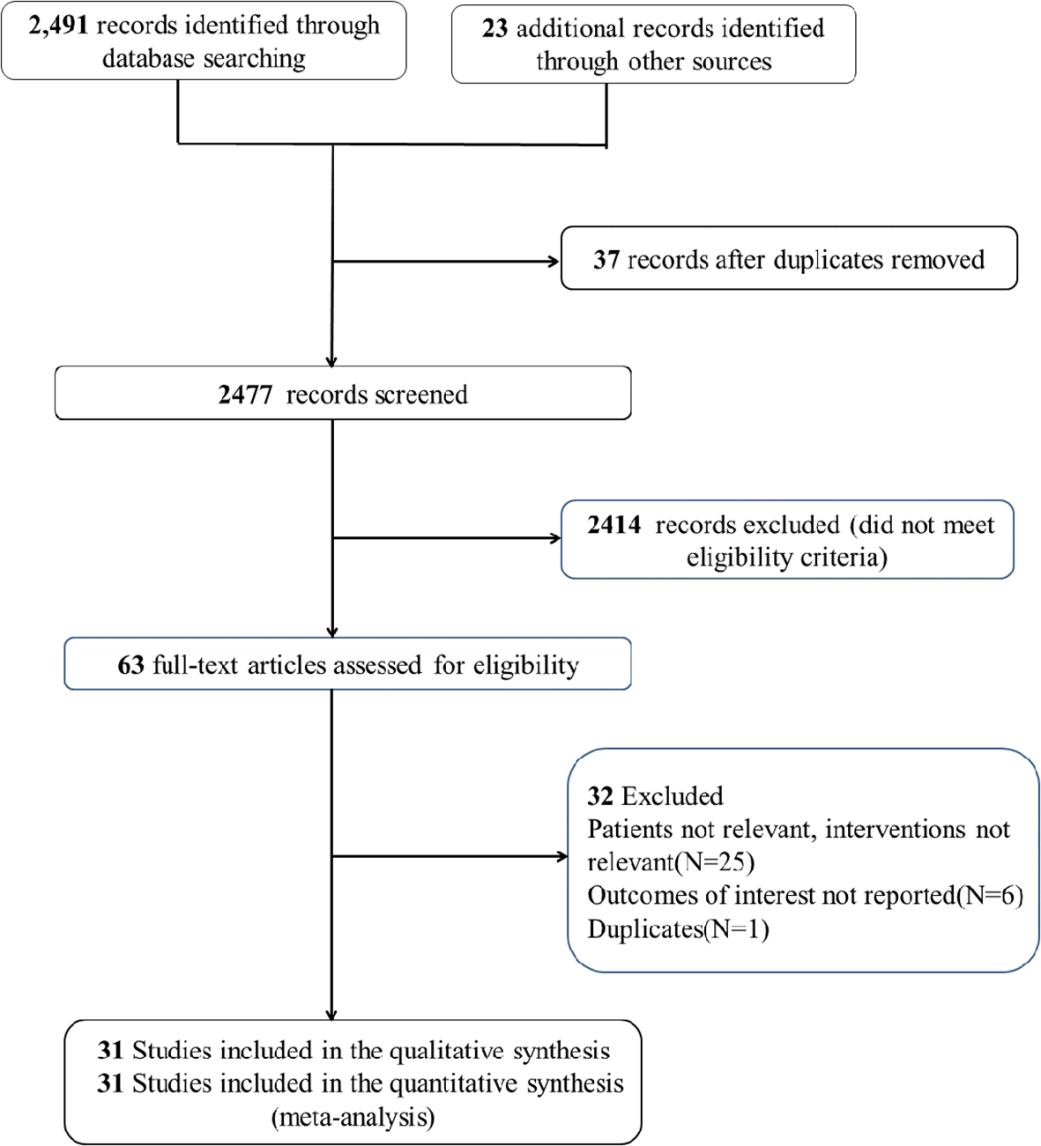
Flowchart of selecting studies for review.

An extensive search was performed in the PubMed, EMBASE, and Cochrane CENTRAL databases for studies published up to 1 June, 2023. A combination of MeSH/Emtree terms and title/abstract keywords was used, focusing on “statins” and “breast cancer” (detailed in **Stable 1**). Included studies met these criteria: (1) involved adult patients diagnosed with breast cancer; (2) compared groups with and without statin use; (3) examined the relationship between statins and breast cancer mortality or recurrence; (4) provided detailed data on hazard ratios (HR) and 95% confidence intervals (CI) for outcomes such as recurrence, breast cancer-specific mortality, and all-cause mortality; and (5) were observational studies written in English. Exclusions were made for case reports, reviews, and preprints (search process illustrated in **Figure 1**).

### Data extraction

Key information was extracted from selected studies, including the first author, publication year, study methodology, duration of follow-up, sample size, average age of participants, details of the statin intervention, and study outcomes.

### Statistical analysis

The data for this meta-analysis were processed using STATA 12.0 software. To assess the impact of statins on breast cancer prognosis, HR and 95% CI from selected studies were extracted. Study variations were evaluated using the Q test and I^2^ index. A *P*-value less than 0.05 and I^2^ greater than 50% indicated significant heterogeneity, prompting the use of random-effects models for combined outcomes. Additionally, sensitivity analysis, Egger’s test, and funnel plots were employed to assess the robustness and potential publication bias of the findings.

## Results

### Study selection and characteristics

A total of 31 studies (13–43) were included in this meta-analysis, encompassing data from 261,834 female breast cancer patients. Among these, five studies (16, 23, 27, 28, 32) provided data on the impact of statin use prior to diagnosis on breast cancer-specific mortality, while another five (16, 23, 28, 32, 37) discussed its effects on all-cause mortality. Additionally, 13 studies (13, 18, 21, 25, 30, 34–36, 38, 40–43) reported on the influence of statin use after diagnosis on breast cancer recurrence. A further 12 articles (14, 16–18, 21, 22, 26, 28, 29, 31, 33, 39) and 19 studies (13–22, 24, 26–31, 36, 39) provided results regarding the effects of post-diagnosis statin use on overall mortality and breast cancer-induced mortality, respectively. The primary details from these studies are summarized in **Table 1**.

**Table 1:** Characteristics of the included studies in the meta-analysis of statin vs non-statin use in adult breast cancer patients.

Study	Study design	Patient characteristics	Median Follow-up	Sample size	Mean age (years)	Experimental intervention	Outcomes
Ahern et al. 2021	Prospective cohort study	Residents in Denmark diagnosed with stage I–III invasive breast cancers.	6.8 years	18,769	NA	Patients prescribed a statin were assumed to be exposed (i.e., statin users), whereas patients who were not prescribed a statin were assumed to be unexposed to statins (i.e., non-users).	Breast cancer recurrence risk
Boudreau et al. 2014	Cohort study	>18 years; residing in Washington State SEER; stage I/II breast cancer; no bilateral disease.	6.3 years	4,216	63	Statins, angiotensin converting enzyme inhibitors (ACEI), beta blockers (BB), calcium blockers, and diuretics were the exposures of interest.	Recurrence or second primary breast cancer
Chae et al. 2011	NA	Diagnosed with stage II/III breast cancer.	55 months	703	59.11	Statin and ACEI or angiotensin receptor blockers (ARB) users were arbitrarily defined as patients who took the medication in non-evident disease (NED) stage for at least 6 months.	Disease-free survival and overall survival
Choi et al. 2021	Retrospective cohort study	Had undergone surgery for breast cancer.	NA	7,452	NA	Exposure was defined as a recorded prescription of each drug within 12 months before diagnosis of breast cancer.	Overall survival
Harborg et al. 2020	Nationwide, population-based cohort study	Postmenopausal women diagnosis of stage I–III ER+breast cancer.	Began 7 months after diagnosis to recurrence, death, emigration, 5 years elapsed or the end of available follow-up data on 25 September, 2018	14,773	NA	Ascertained incident statin exposure (≥1 prescription post-diagnosis) from the Danish National Prescription Registry and modeled statins as a time-varying exposure lagged by 6 months.	5-year breast cancer recurrence
Hosio et al. 2020	Observational cohort study	Women with type 2 diabetes mellitus (T2DM) and diagnosed with breast cancer in Finland.	4.6 years	3,533	72	A patient who first purchased a statin ≥180 days before breast-cancer diagnosis was classified into the group of statin users.	Breast cancer-related mortality rate
Inasu et al. 2022	Cohort study	Breast cancer diagnosed.	8.6 year	360	NA	For a patient to be defined as a statin user in the analyses, the patient had to fill a prescription of statins.	Risk of breast cancer recurrence
Kwan et al. 2008	Cohort study	Diagnosed from the ages of 18–79 years with a first primary breast cancer (stage I 1 cm, II, or IIIA).	2.60 years	1,945	62.2	Information on statin use was obtained from the KPNC pharmacy database.	Risk of breast cancer recurrence
Lu et al. 2021	Population-based cohort study	Newly diagnosed female breast cancer patients (ICD-9-CM 174) who were aged ≥20 years from Taiwan.	NA	6,110	57.8	Patients with statin treatment within 730 days (2 years) after breast cancer’s initial date were defined as the statin cohort to avoid immortal time bias.	Occurrence of primary breast cancer
Sakellakis et al. 2016	Retrospective study	Female patients with stage I, II, or III breast cancer who had been surgically treated and who had subsequently received at least one adjuvant chemotherapy.	NA	610	56 years	Whether they were receiving a statin on a chronic basis or not. Information regarding statin use was obtained during the initial visit.	Risk of recurrence
Sim et al. 2022	Retrospective study	Diagnosed with breast cancer, and stage I–III invasive cancers were included.	8.67 years	7,858	NA	Statin use was defined as use after surgery	Risk of recurrence; Disease-specific survival
Bjarnadottir et al. 2020	Population-based prospective cohort study	All residents of Malmö by 1 January, 1991 and born between 1926 and 1945.	NA	910	56.4	Information on statin use was obtained from the Swedish Prescribed Drug Register from July 2005, when the registry was initiated, through 2016.	Breast cancer-specific mortality
Borgquist et al. 2019	Nationwide retrospective cohort study	Swedish women diagnosed with breast cancer.	61.6 months	20,559	64	Statin use was obtained from the Swedish Prescription Registry. Patients were classified as exposed to statins if they had at least one statin prescription logged in their pharmaceutical records. Women prescribed a statin at least once were classified as statin users, whereas others were assumed to be unexposed to statins and therefore classified as non-users.	Breast cancer-specific and overall mortality
Botteri et al. 2013	Retrospective study	Postmenopausal women diagnosed and operated for early primary triple-negative breast cancer (TNBC).	NA	800	62 years in BB users and 59 years in non-users	The only purpose of limiting the analysis to postmenopausal women was to avoid high heterogeneity between BB users and non-users. Patients were divided into two groups: the BB user group, which included patients who were using any BB at the moment of their diagnosis of TNBC, and the BB non-user group, which included all the other patients.	Risk of breast cancer-related recurrence, metastasis, and breast cancer mortality
Brewer et al. 2013	NA	Diagnosed with stage I–III of inflammatory breast cancer (IBC).	2.9 years	723	NA	Statin users were defined as being on statins at the initial evaluation. Based on Ahern et al.’s statin classification, clinical outcomes were compared by statin use and type (weakly lipophilic to hydrophilic (H-statin) vs lipophilic statins (L-statin)).	Median progression-free survival, overall survival, and disease-specific survival
Desai et al. 2015	Cohort study	Postmenopausal women aged 50–79 years, and it was classified into early (in situ and local)- versus late (regional and distant)-stage disease.	11.5 years	10,474	63.4	Statin use was defined as any HMG-CoA reductase inhibitor used at baseline or during participation in the Women’s Health Initiative (WHI) prior to diagnosis of breast cancer.	Breast cancer mortality
Haukka et al. 2017	Cohort study	Random sample of the people who had purchased and been reimbursed for at least one prescription of OAD medication (A10B) in the same period.	2.0 years	39,900	NA	Statin and metformin	All-cause and cause-specific mortality
Lacerda et al. 2014	Cohort study	Diagnosed with stage I–III of IBC.	2.5 years	519	49 years	Statins	Local recurrence-free survival
Li et al. 2019	Single-institution, retrospective cohort study	Women diagnosed with operable breast cancer.	70.2 months	1,523	64.9	Statins	Overall and disease-free survival
Menamin et al. 2016	Retrospective cohort study	Newly diagnosed invasive breas t cancer patients.	4 years	1,190	NA	Statin use	Breast cancer-specific mortality
Murtola et al. 2014	Population-based cohort	Breast cancer cases diagnosed in Finland.	3.25 years	31,236	NA	The status of post-diagnostic statin use was updated prospectively for each year of follow-up since breast cancer diagnosis. The study participant was categorized as the statin user only for the years with recorded statin purchases, regardless of the amount.	Disease-specific mortality
Nowakowska et al. 2021	Cohort study	Women aged ≥66 years who had stage I–III breast cancers were identified.	4.4 years	23,192	NA	Statin use, defined as the initiation of statin therapy in the 12 months after breast cancer diagnosis.	Overall survival and breast cancer-specific survival
Scott et al. 2023	Cohort study	Eligible women were all those with a first primary breast cancer diagnosed.	4.51 years	14,976	NA	Statins	Breast cancer-specific mortality
Shaitelman et al. 2017	Cohort study	Women with invasive, non-metastatic TNBC.	75.1 months	869	NA	Statin use, defined as ever use during treatment vs never use	Median overall survival, distant metastases-free survival, and local-regional recurrence-free survival
Smith et al. 2016	Cohort study	Women with stage I–III breast cancers.	4.9 years	4,243	66	De novo post-diagnostic statin exposure was identified from prescriptions dispensed between breast cancer diagnosis and end of follow-up (death or 31 December, 2012, whichever occurred first).	Breast cancer-specific or all-cause mortality
Smith et al. 2017	Cohort study	Women with stage I–III breast cancers.	NA	6,314	71	Patients with de novo post-diagnostic statin use were excluded from analyses to determine the effect of statin use in patients with pre-diagnostic use.	Breast cancer-specific and all-cause mortality
Takada et al. 2023	Cohort study	Diagnosed with breast cancer, with a primary lesion of 2 cm or less identified by preoperative imaging, and who underwent surgery without preoperative chemotherapy.	NA	719	58 years	Statins	Recurrence-free survival or overall survival
Chang et al. 2023	Cohort study	Diagnosed with breast cancer from Taiwan.	4.10 years	14,902	65.06	Patients receiving statins within 6 months before diagnosis of breast cancer were compared with those not receiving statins.	Death, heart failure, and arterial and venous events
Cardwell et al. 2015	Nested case-control cohort study	Diagnosed with breast cancer.	5.7 years	17,880	NA	Dose-response analyses were conducted, with a patient considered to be a non-user before 6 months after first use, a short-term user between 6 months after first use and 6 months after the 12th prescription (or 365 daily defined doses), and a longer-term user after this time.	Breast cancer-specific and all-cause mortality
Tryggvadottir et al. 2018	Population-based prospective cohort study	Women diagnosed with a primary breast cancer.	7.0 years	985	61	Medications were coded according to the Anatomic Therapeutic Chemical classification system codes; code C10AA was used for statins, 92.5% were using lipophilic statins (simvastatin or atorvastatin).	Overall survival
Kim et al. 2021	Population-based cohort study	Aged 45 to 70 years old.	7.6 years	3,591	NA	Women aged 45 to 70 years old who had taken statins for at least 6 months were compared to statin non-users of the same age.	Breast cancer-related mortality after diagnosis

NA, not acquired.

### Outcomes

**Figure 2:**
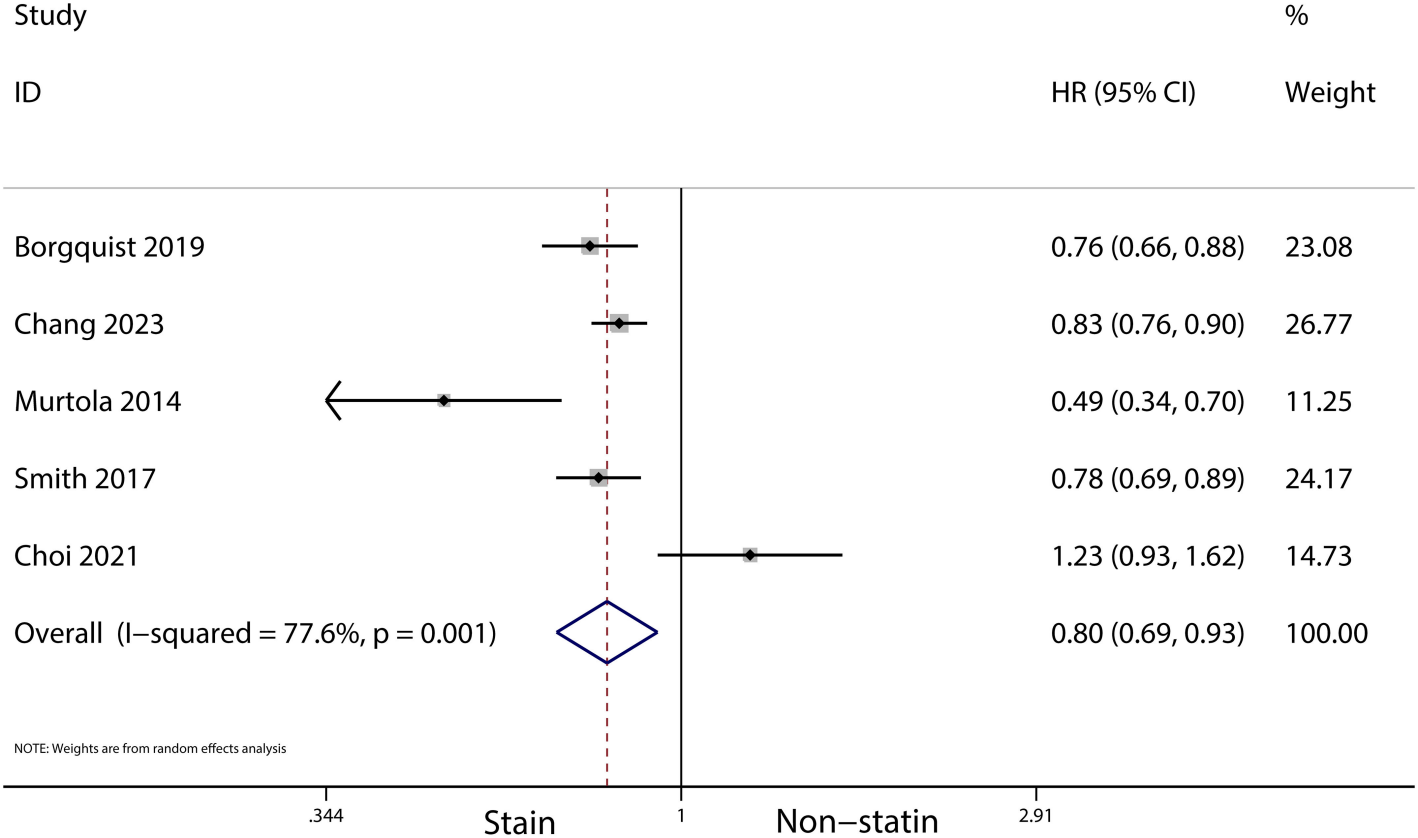
Forest plot of the HR of this meta-analysis for the overall mortality between statin and non-statin use in patients with prior to breast cancer.

**Figure 3:**
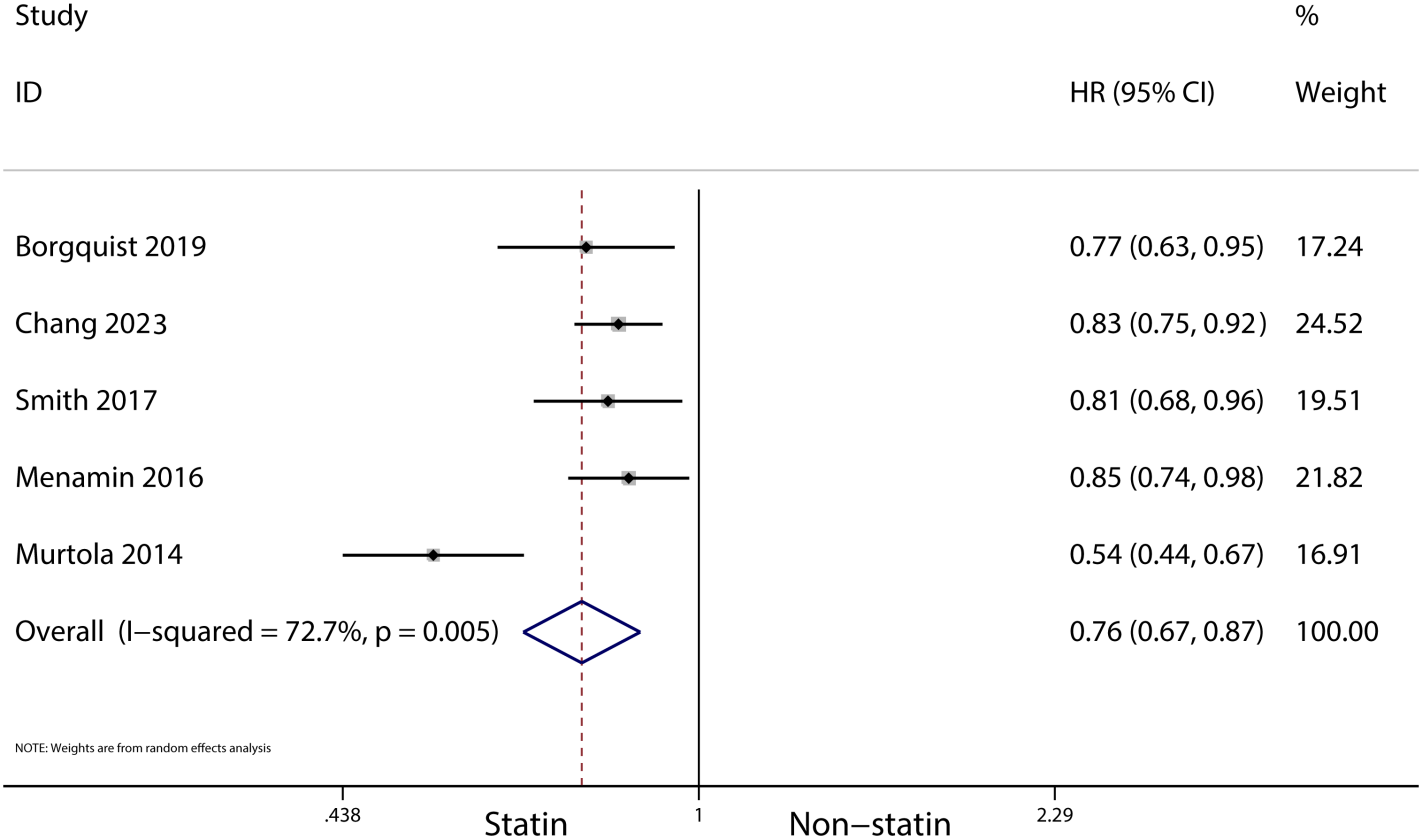
Forest plot of the HR of this meta-analysis for breast cancer-specific mortality in patients with prior to diagnosis breast cancer.

**Figure 4:**
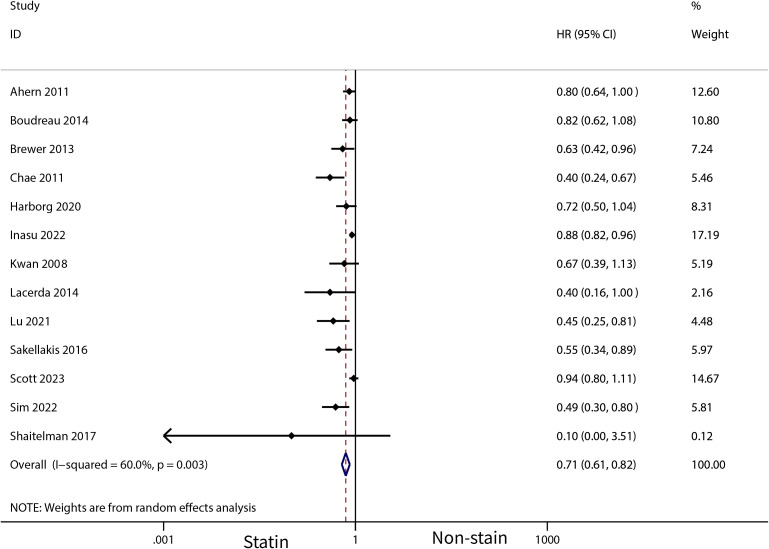
Forest plot of the HR of this meta-analysis for breast cancer recurrence in patients with post-diagnosis breast cancer.

**Figure 5:**
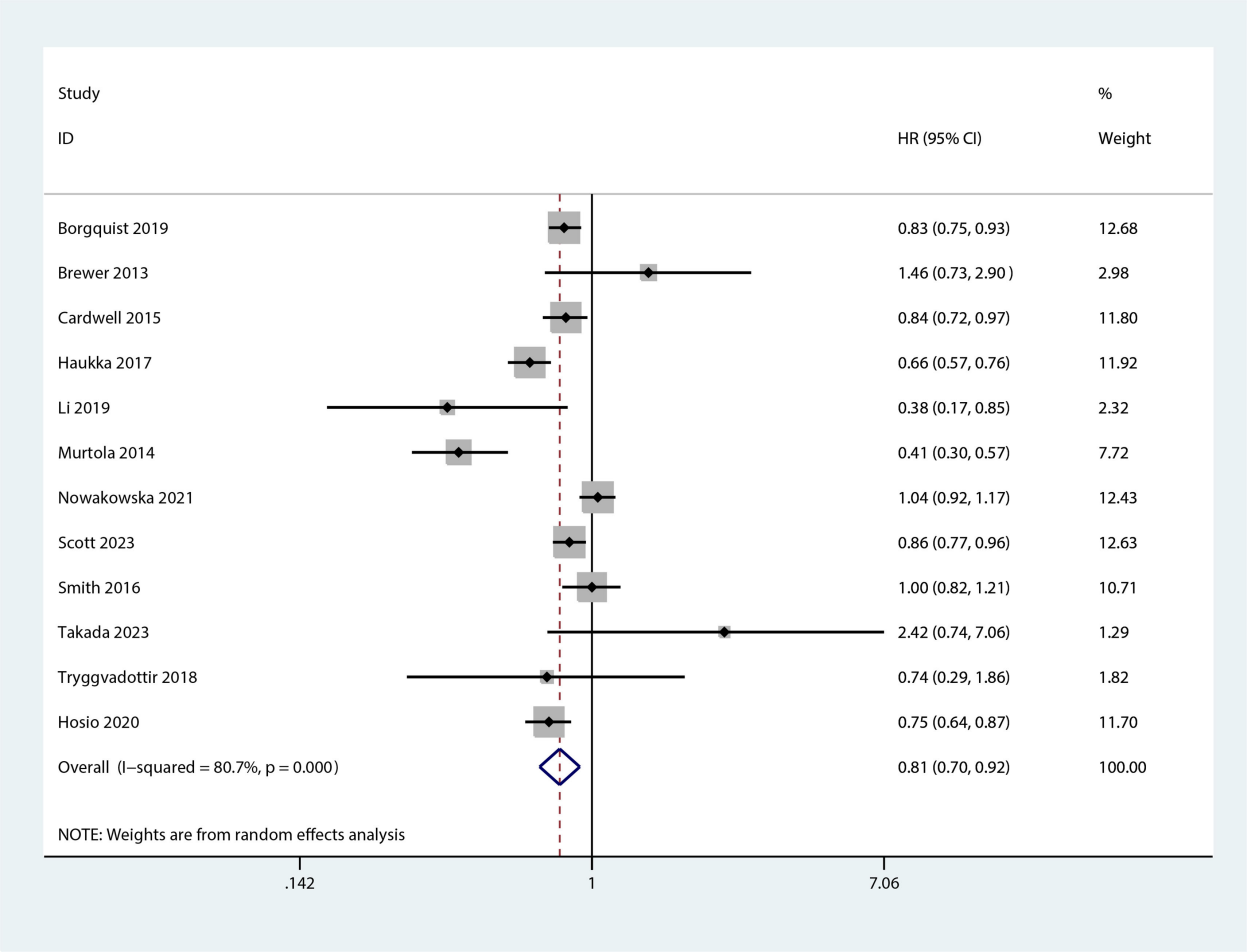
Forest plot of the HR of this meta-analysis for the overall mortality between statin and non-statin use in patients with post-diagnosis breast cancer.

**Figure 6:**
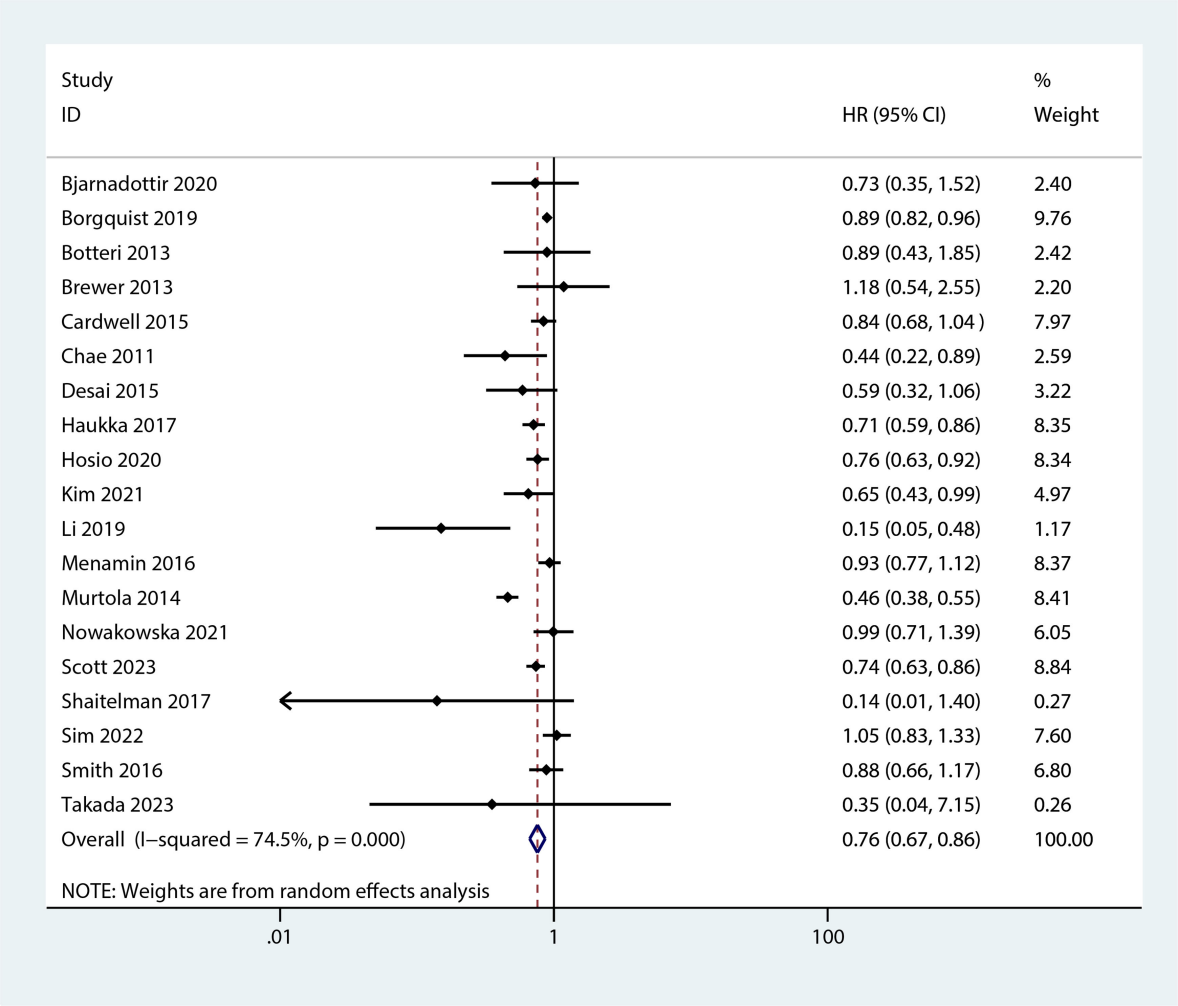
Forest plot of the HR of this meta-analysis for breast cancer-specific mortality prior to diagnosis in patients with post-diagnosis breast cancer.

The meta-analysis revealed that statin use, compared to non-use, was associated with a lower risk of overall mortality (HR, 0.8; 95% CI, 0.69–0.93; I^2^ = 77.6%; *P* = 0.001; **Figure 2**) and breast cancer-specific mortality prior to diagnosis (HR, 0.76; 95% CI, 0.67–0.87; I^2^ = 72.7%; *P* = 0.005; **Figure 3**). Post-diagnosis statin use also appeared to decrease breast cancer recurrence (HR, 0.71; 95% CI, 0.61–0.82; I^2^ = 60%; *P* < 0.003; **Figure 4**), overall mortality (HR, 0.81; 95% CI, 0.70–0.92; I^2^ = 80.7%; *P* < 0.001; **Figure 5**), and breast cancer-specific mortality (HR, 0.76; 95% CI, 0.67–0.86; I^2^ = 74.5%; *P* < 0.001; **Figure 6**). Sensitivity analysis indicated consistent results across studies (refer to **Supplementary Figures 2**, **5**, **8**, **11**, **14**). Egger’s tests and funnel plots suggested no significant publication bias (Egger’s tests: **Supplementary Figure 3**, *P* = 0.821; **Supplementary Figure 6**, *P* = 0.229; **Supplementary Figure 9**, *P* < 0.01; **Supplementary Figure 12**, *P* = 0.698; **Supplementary Figure 15**, *P* = 0.148; and funnel plots: **Supplementary Figures 1**, **4**, **7**, **10**, **13**).

**Figure 7:**
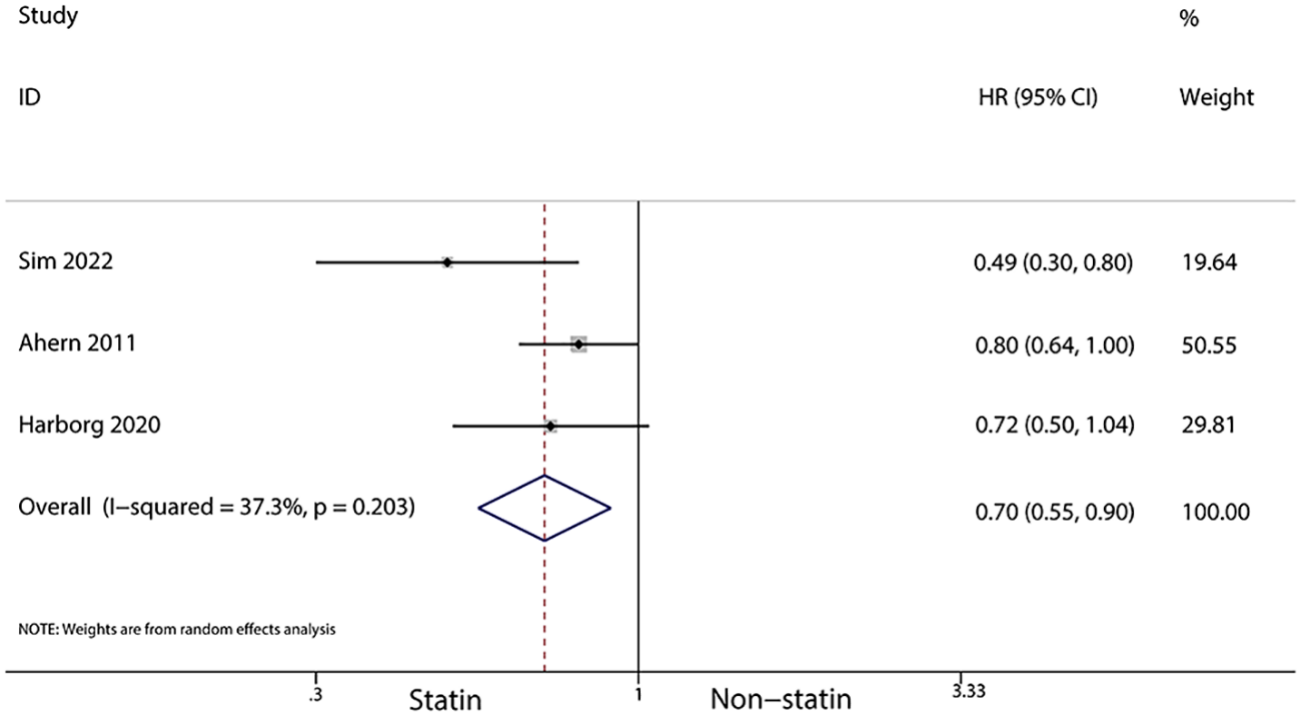
Forest plot of the HR of this meta-analysis of statins for more than a year after diagnosis for breast cancer recurrence in patients with breast cancer.

**Figure 8:**
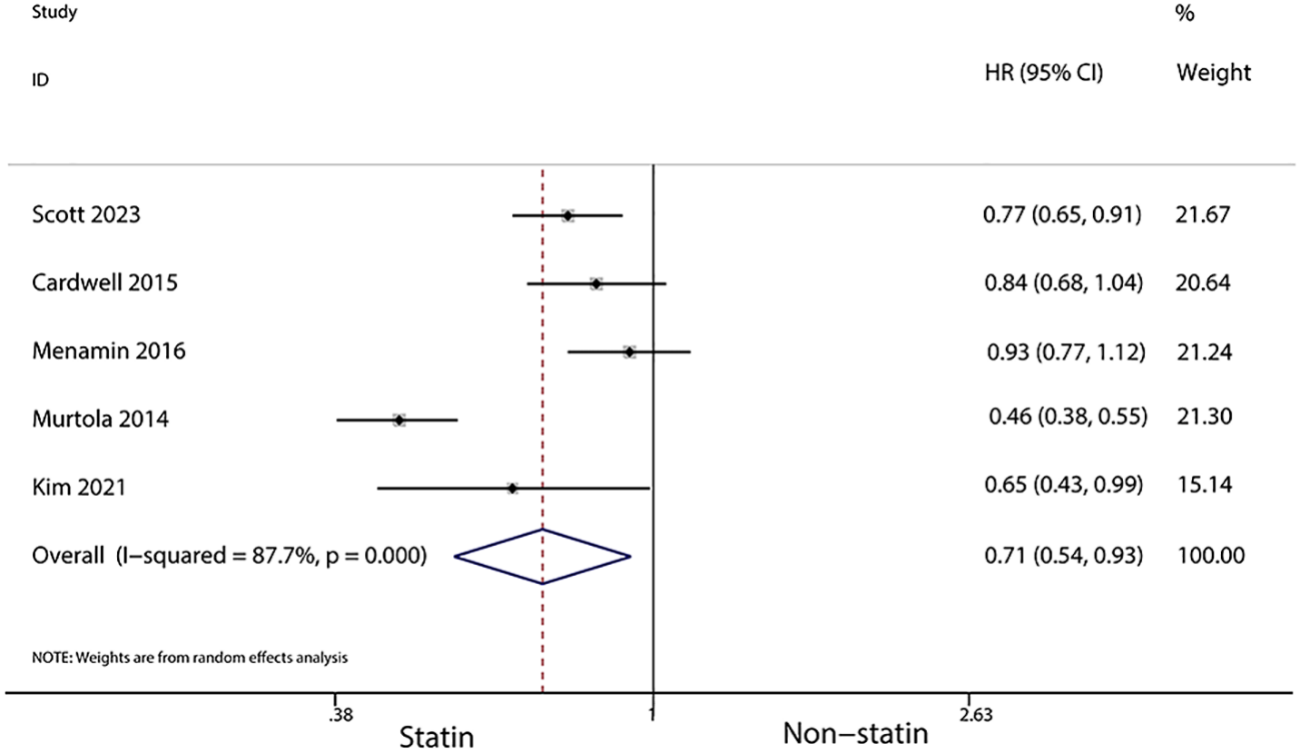
Forest plot of the HR of this meta-analysis of statin use for over six months post-diagnosis for cancer-related death in patients with breast cancer.

For patients using statins for more than a year after diagnosis, a further reduced risk of recurrence was noted (HR, 0.7; 95% CI, 0.55–0.90; I^2^ = 37.3%, *P* = 0.203; **Figure 7**). Additionally, statin use for over six months post-diagnosis was linked with a lower risk of cancer-related death (HR, 0.71; 95% CI, 0.54–0.93, I^2^ = 87.7%, *P* < 0.001; **Figure 8**). Murtola et al. (28) observed that increased intensity of statin use post-diagnosis was associated with greater reductions in cancer-related deaths, especially in patients with metastatic tumors. The lowest intensity of statin use (14–183 defined daily doses/year) correlated with the smallest reduction in cancer deaths in metastatic patients (HR, 0.66; 95% CI, 0.4–1.09), while medium (184–300 defined daily doses/year) and high intensity (301 or more defined daily doses/year) were associated with greater reductions in mortality (HR, 0.43; 95% CI, 0.2–0.9 and HR, 0.42; 95% CI, 0.19–0.94, respectively).

## Discussion

This meta-analysis synthesized data from 31 studies involving 2,611,014 women diagnosed with breast cancer. It focused on assessing the impact of statins on the risk of overall mortality, breast cancer-specific mortality, and the recurrence of breast cancer. Our findings suggest a beneficial effect of statins, both prior to and after diagnosis, in reducing overall mortality, breast cancer-specific mortality, and the recurrence of the disease. These results are in line with a previous study (44) that reviewed 23 articles involving 178,712 breast cancer patients, which also suggested that statin use could decrease overall mortality, disease-induced mortality, and recurrence in women with breast cancer. However, the earlier meta-analysis (44) was limited to post-diagnosis statin use and did not include data on pre-diagnosis use. Other research (45) has indicated that statins may reduce disease-induced mortality and recurrence in breast cancer patients, but did not differentiate between pre- and post-diagnosis use of statins, and found no significant impact on the risk of developing breast cancer. Additionally, a study (46) involving seven studies and 197,048 women with breast cancer indicated that statins might reduce both disease-induced and overall mortality. In addition, a randomized controlled study (47) showed that statin use after diagnosis was associated with a reduction in all-cause mortality (HR, 1.16; 95% CI, 0.7–1.91). Furthermore, there is evidence (48) suggesting an enhanced effect on breast cancer reduction when pitavastatin is used alongside neoadjuvant chemotherapy protocols.

Ongoing research is examining the mechanisms through which statins may decrease mortality in breast cancer patients. The mevalonate pathway, a common feature in cancerous lesions, is upregulated and can be inhibited by statins, exerting an anti-cancer effect. Statins also have an association with ferroptosis (49) and can impair autophagy flux, leading to breast cancer cell death via the ERK1/2 and Akt pathways (50, 51). Preclinical studies suggest that statins can reduce the activity of breast cancer cells, limit their proliferation, and induce apoptosis (52). Atorvastatin may enhance antitumor immunity by inhibiting extracellular vesicles programmed death-ligand 1 (PD-L1) (53). Furthermore, altering cholesterol abundance in cancer cells could affect cellular membrane fluidity and signal transduction, influencing cancer progression (54). The role of statins in three estrogen receptor-positive cancers (breast, endometrial, and ovarian) is currently under investigation, with studies reporting a beneficial role in reducing incidence and mortality (55). In patients with estrogen-dependent breast cancer, post-diagnostic statin use has been linked to reduced cancer-specific mortality (56), potentially by disrupting hormone receptor-related pathways (57). The combination of metformin and simvastatin is reported to enhance the expression of prolyl hydroxylase 2, decreasing hypoxia-inducible factor 1α expression induced in endothelin 1, leading to more effective antiangiogenic and anti-cancer effects (58).

This meta-analysis has some limitations. Firstly, the specific types of statins used were not clearly detailed in the original studies, and different types of statins might yield different results. Secondly, while we included studies focusing on the effect of statins on the prognosis of breast cancer patients, the dosage of statins varied across these studies. Lastly, the studies included were observational, which may introduce more confounding bias and loss of follow-up.

## Conclusions

In conclusion, the results of this meta-analysis indicate that the use of statins may beneficially impact the recurrence of breast cancer, as well as reduce all-cause mortality and breast cancer-specific mortality, irrespective of whether statins are used before or after a breast cancer diagnosis. However, these findings underscore the necessity for additional high-quality research to more definitively ascertain the effects of statins on the prognosis of women with breast cancer.

## Data availability statement

The original contributions presented in the study are included in the article/**Supplementary Material**. Further inquiries can be directed to the corresponding author.

## Author contributions

XJ: Conceptualization, Investigation, Writing – original draft, Writing – review & editing. YL: Formal Analysis, Methodology, Software, Writing – original draft. ZX: Data curation, Investigation, Methodology, Writing – review & editing. QM: Formal Analysis, Software, Writing – original draft.
