# Supplementary figures and images for: Impact of statin use on breast cancer recurrence and mortality before and after diagnosis: a systematic review and meta-analysis
Source: Front Oncol. 2023 Dec 18;13:1256747. doi: 10.3389/fonc.2023.1256747 (PMC10757972; doi:10.3389/fonc.2023.1256747)

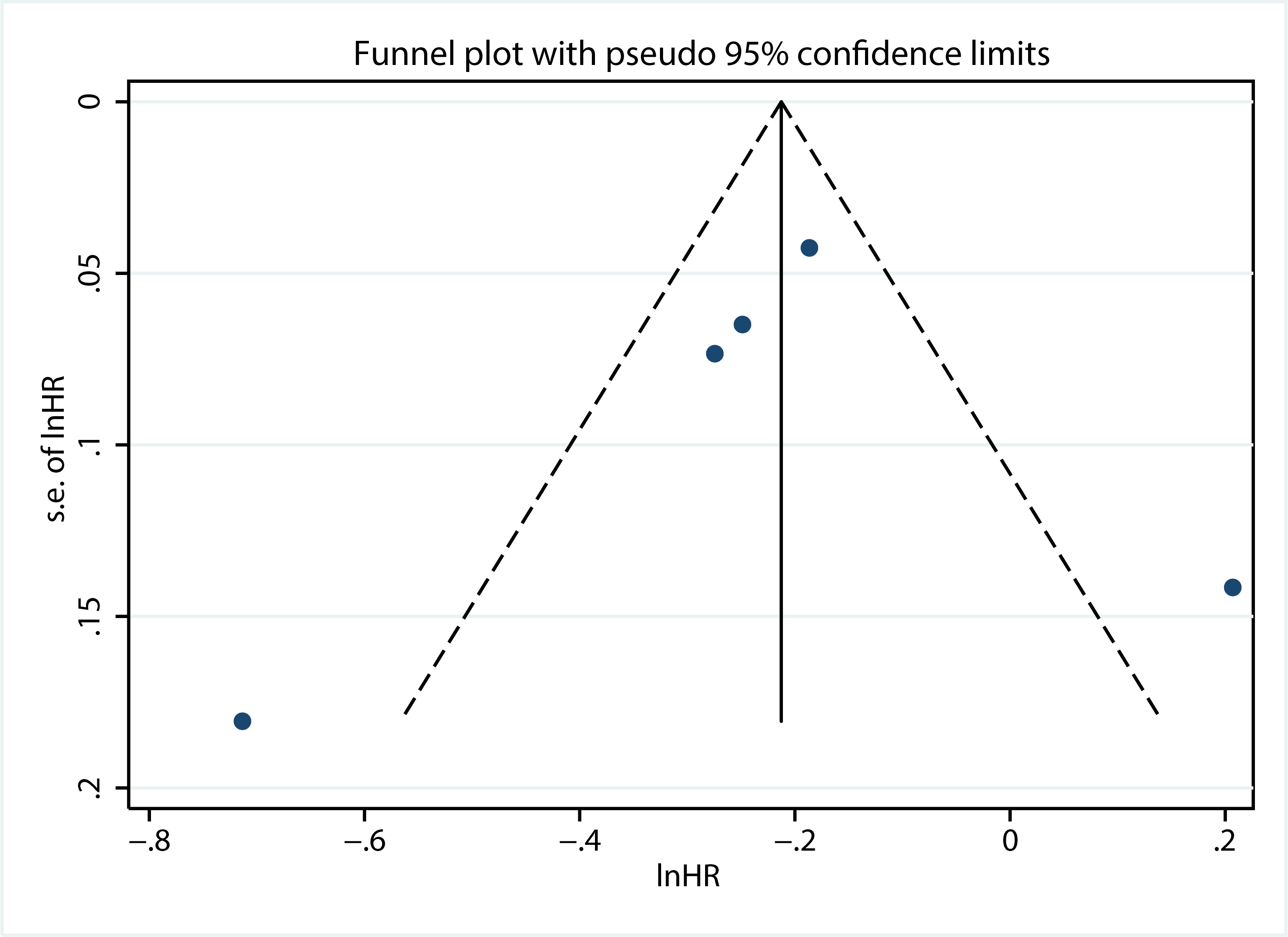

Supplement: Supplementary file 1 [file DataSheet_1.zip › Supplemental materials/SFig 1.tif]

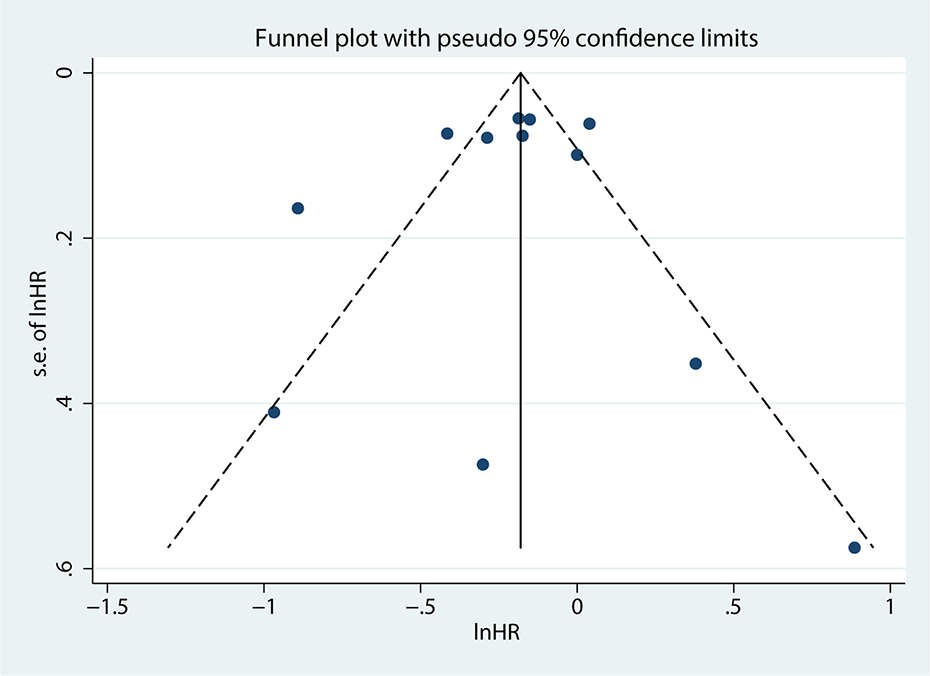

Supplement: Supplementary file 1 [file DataSheet_1.zip › Supplemental materials/SFig 10.tif]

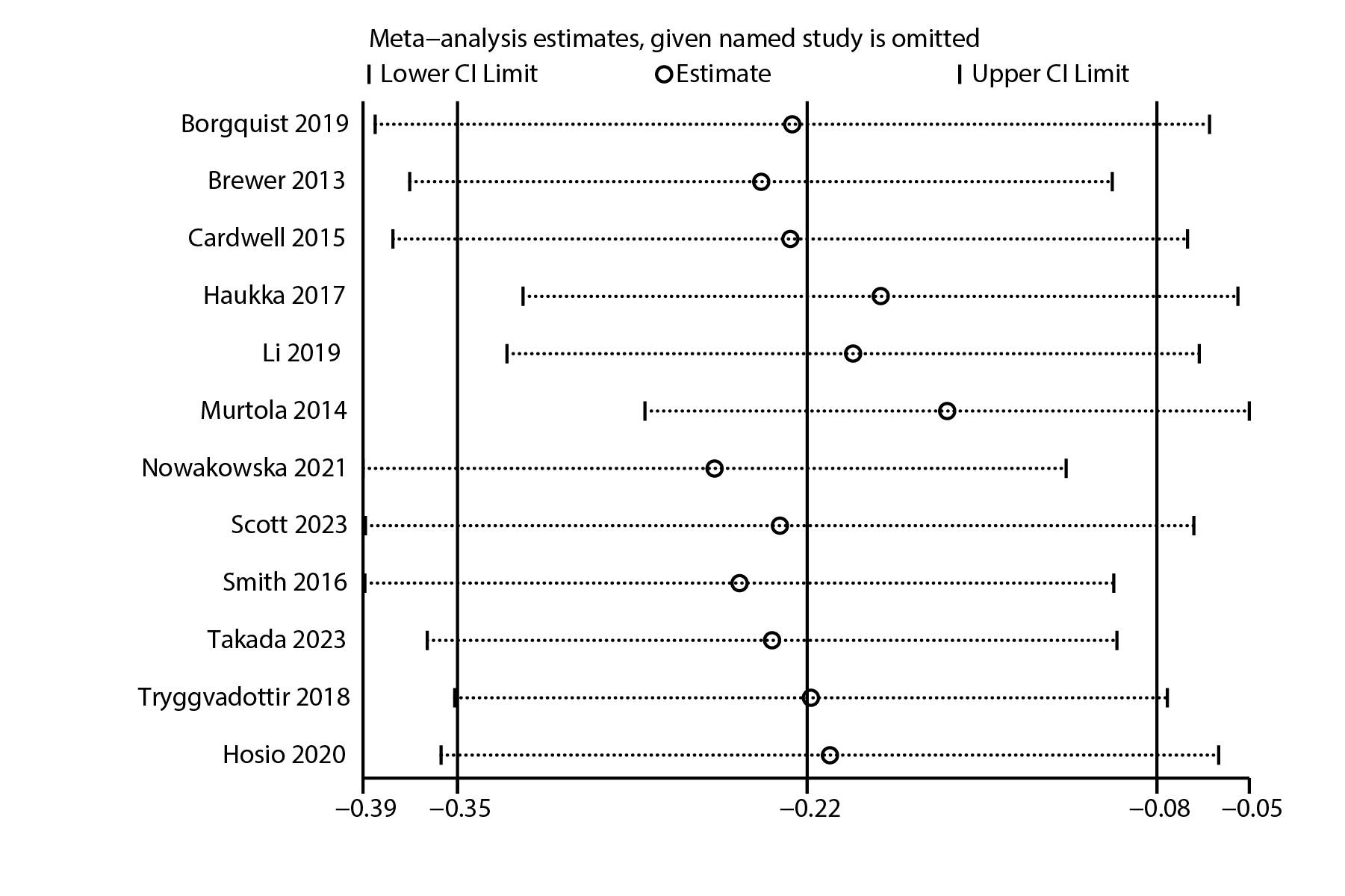

Supplement: Supplementary file 1 [file DataSheet_1.zip › Supplemental materials/SFig 11.tif]

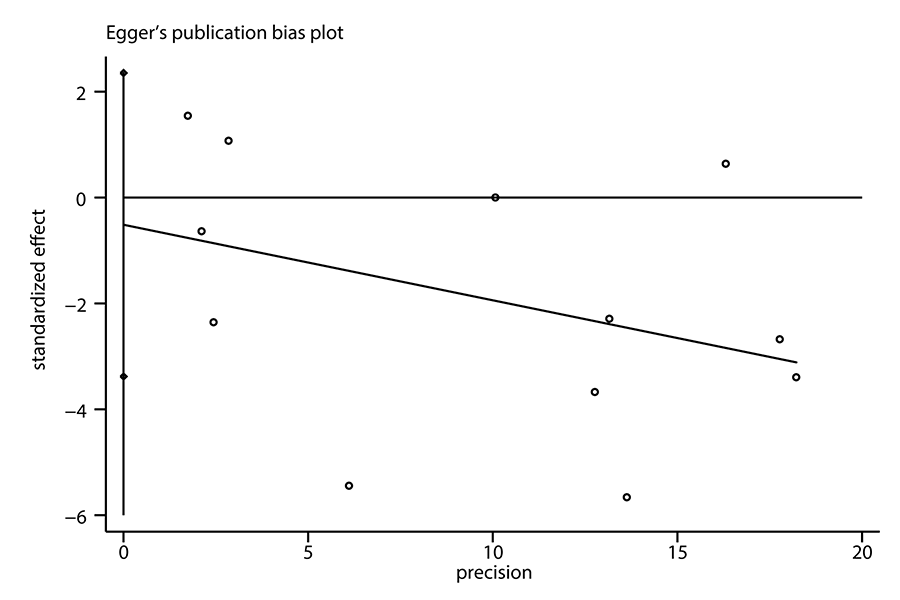

Supplement: Supplementary file 1 [file DataSheet_1.zip › Supplemental materials/SFig 12.tif]

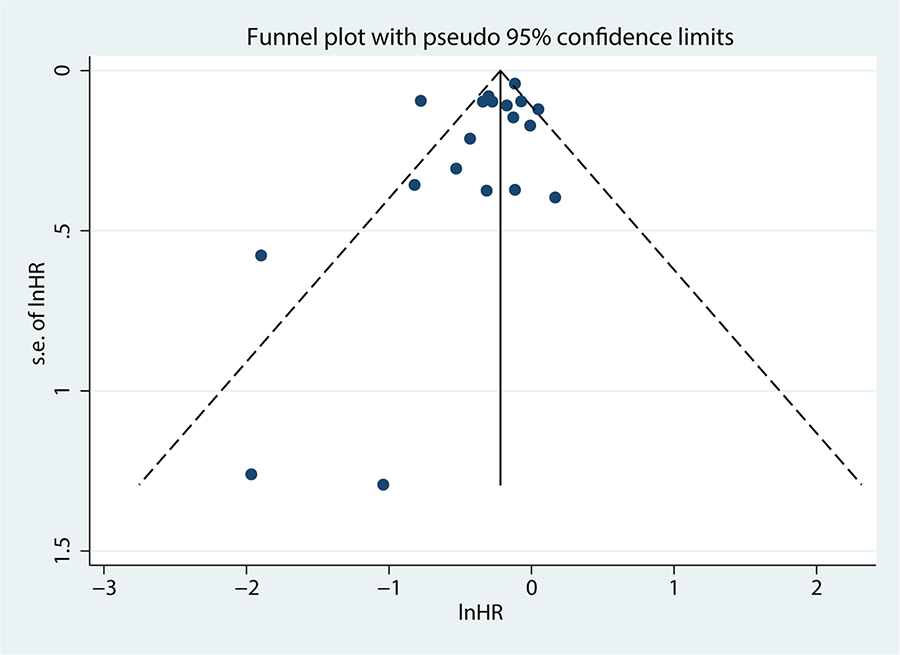

Supplement: Supplementary file 1 [file DataSheet_1.zip › Supplemental materials/SFig 13.tif]

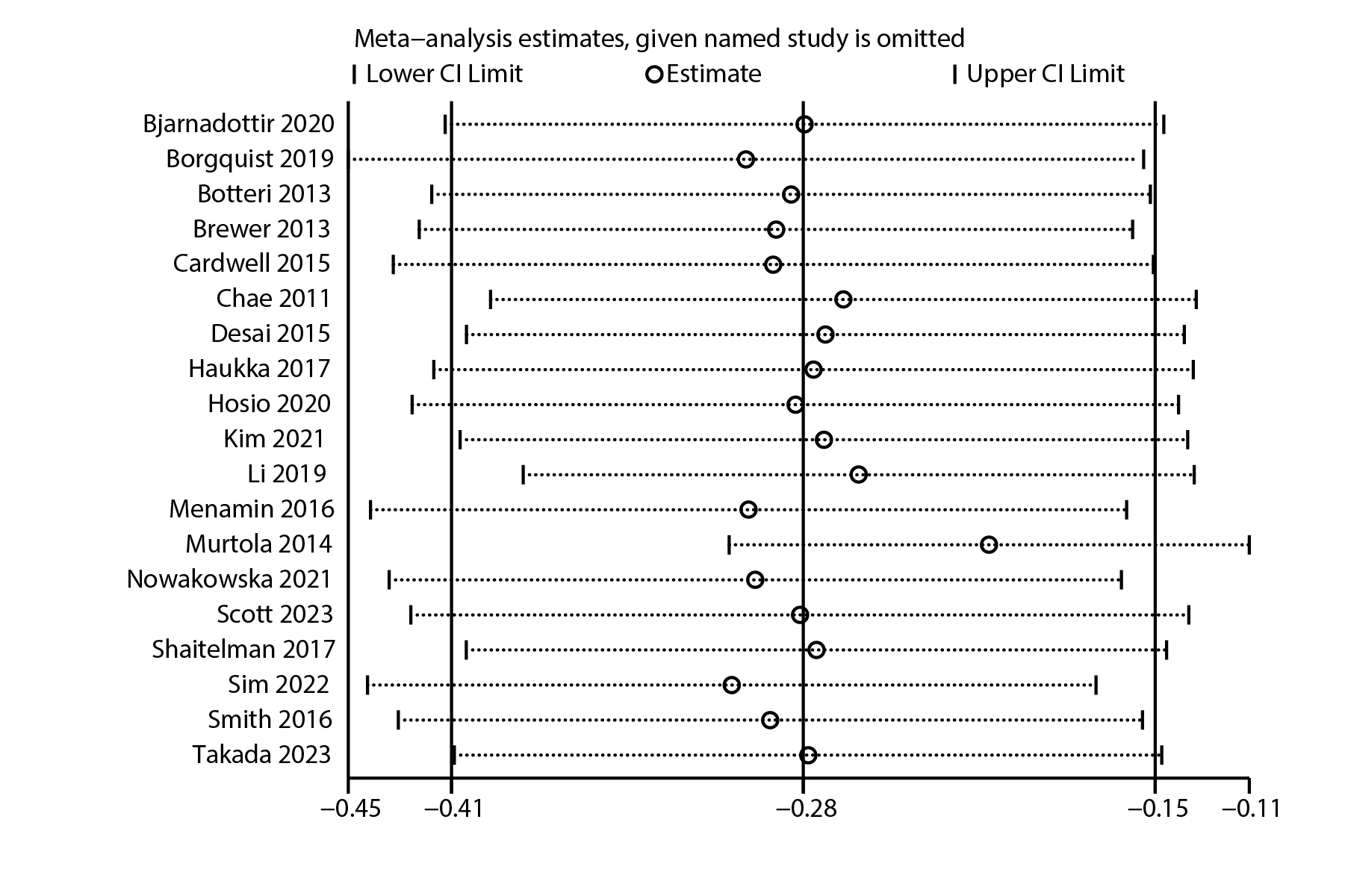

Supplement: Supplementary file 1 [file DataSheet_1.zip › Supplemental materials/SFig 14.tif]

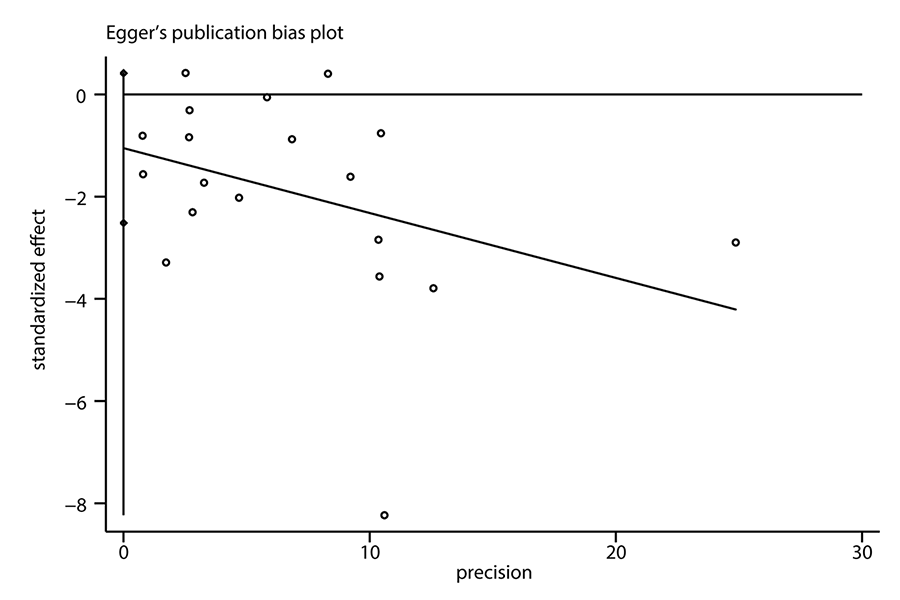

Supplement: Supplementary file 1 [file DataSheet_1.zip › Supplemental materials/SFig 15.tif]

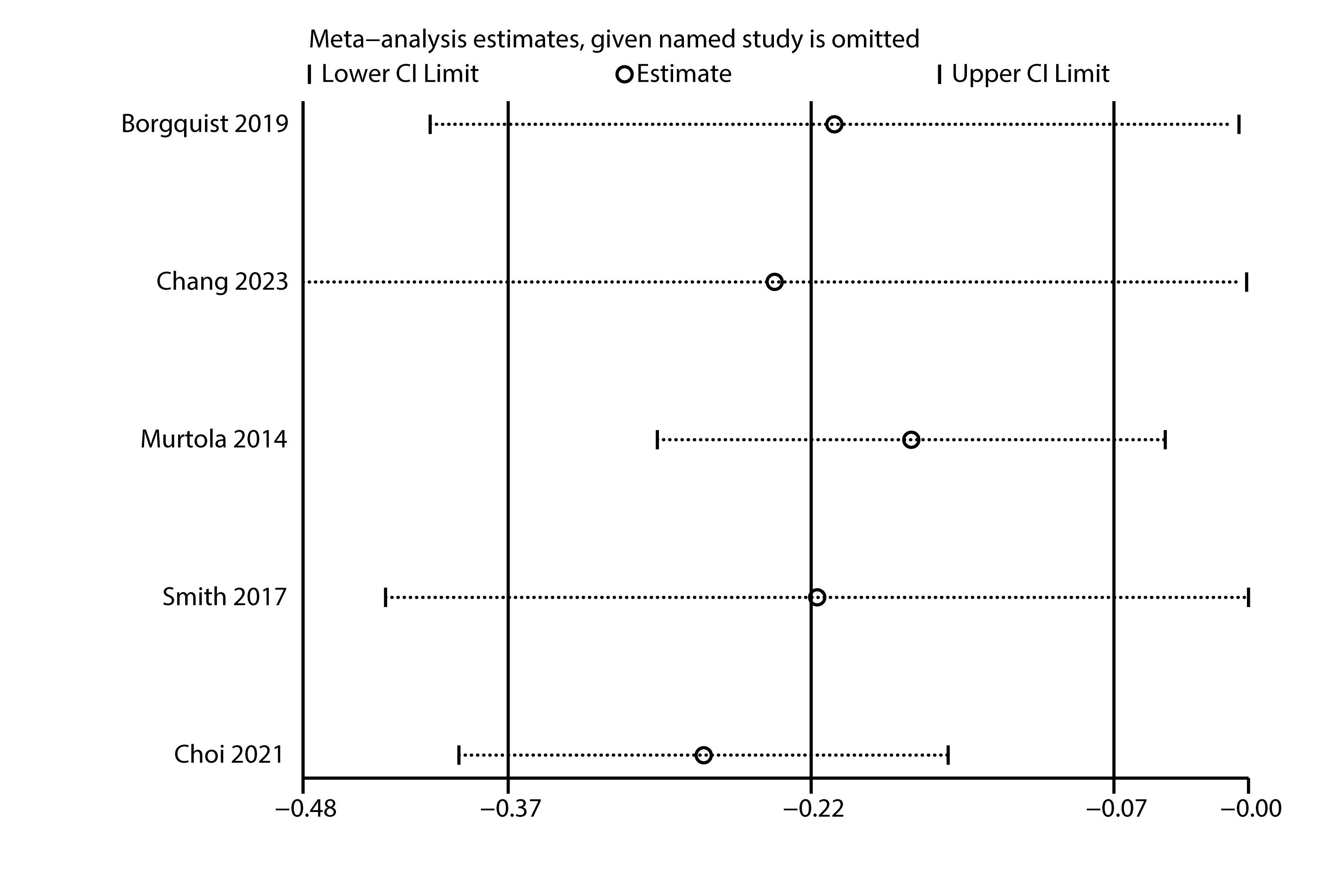

Supplement: Supplementary file 1 [file DataSheet_1.zip › Supplemental materials/SFig 2.tif]

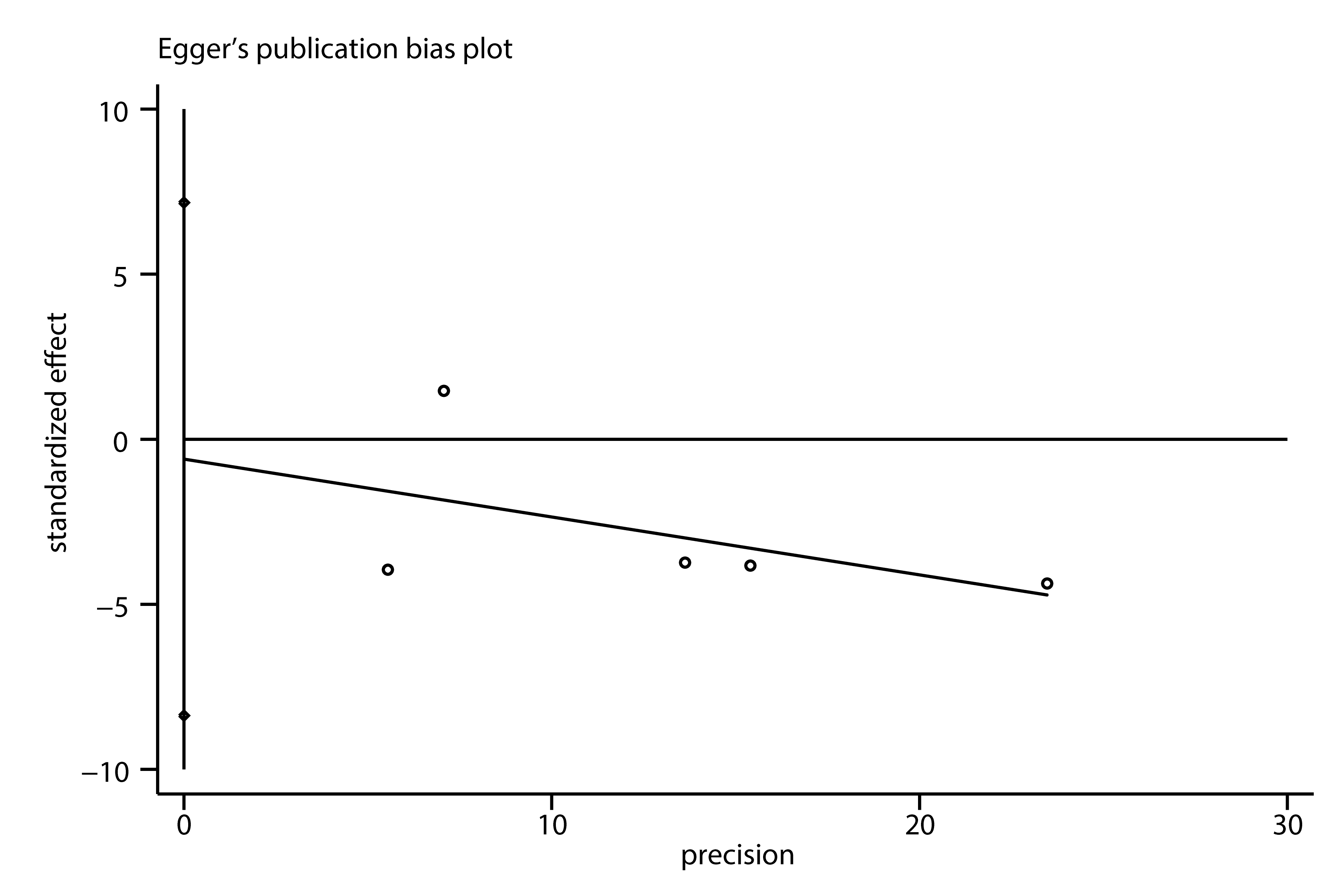

Supplement: Supplementary file 1 [file DataSheet_1.zip › Supplemental materials/SFig 3.tif]

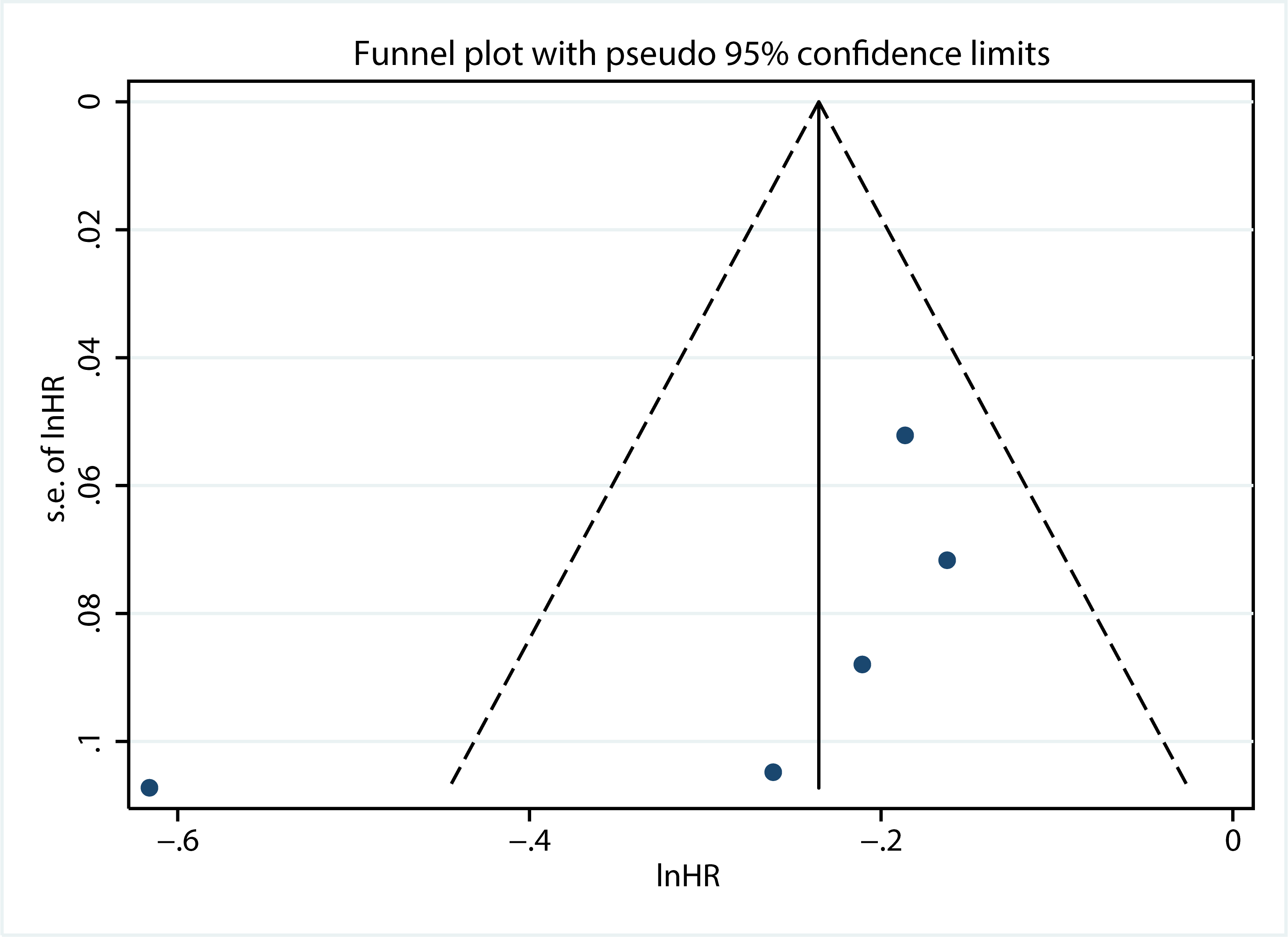

Supplement: Supplementary file 1 [file DataSheet_1.zip › Supplemental materials/SFig 4.tif]

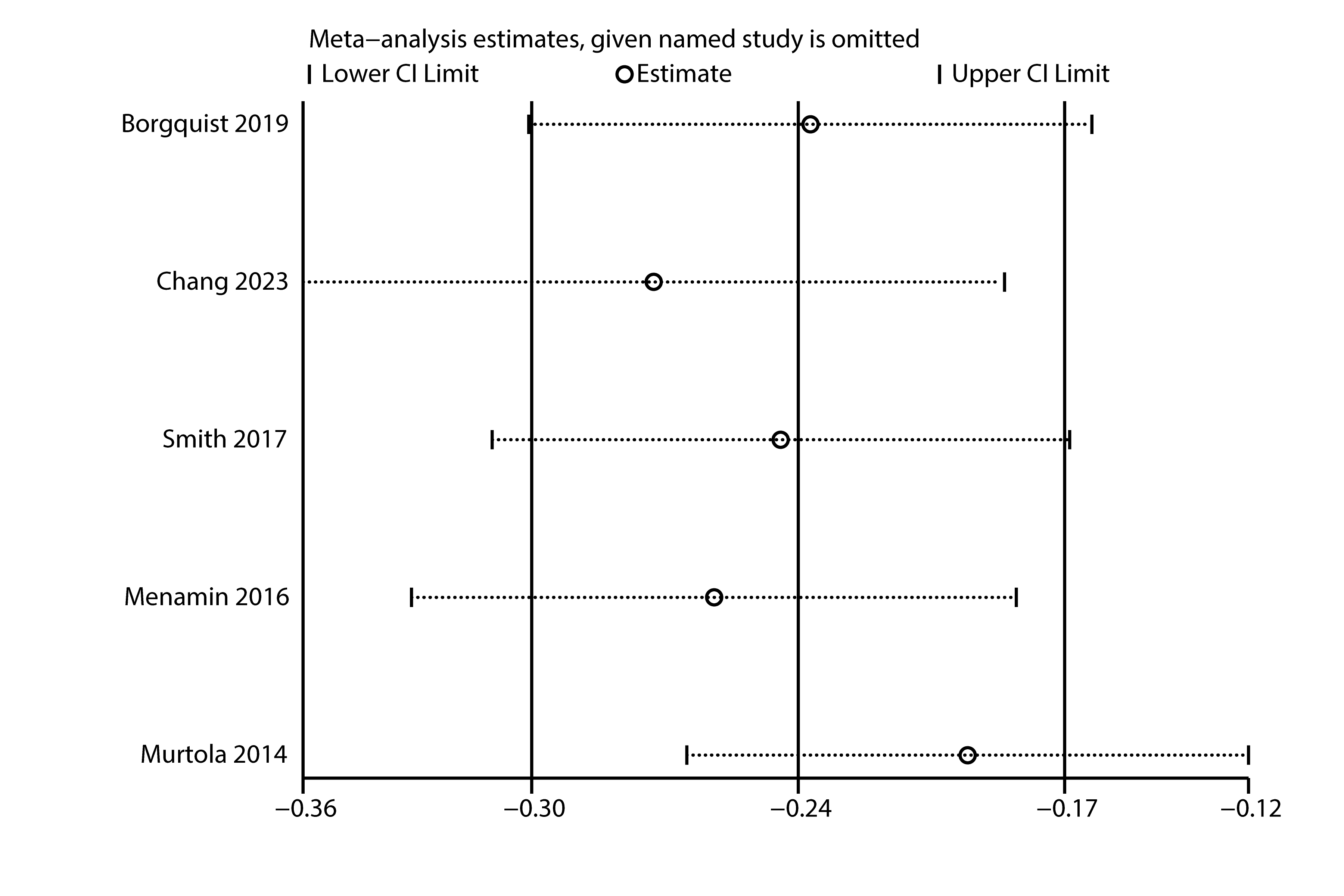

Supplement: Supplementary file 1 [file DataSheet_1.zip › Supplemental materials/SFig 5.tif]

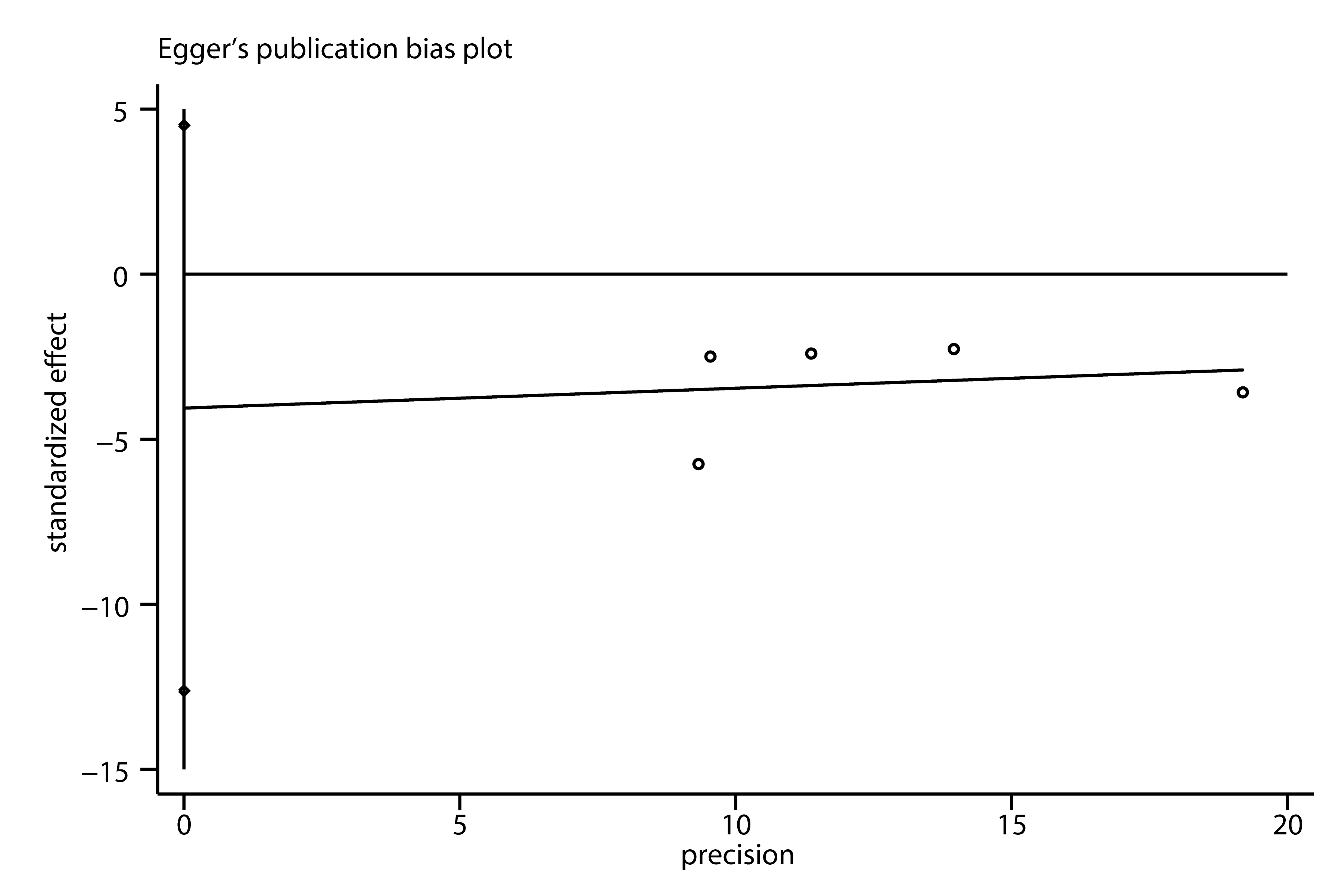

Supplement: Supplementary file 1 [file DataSheet_1.zip › Supplemental materials/SFig 6.tif]

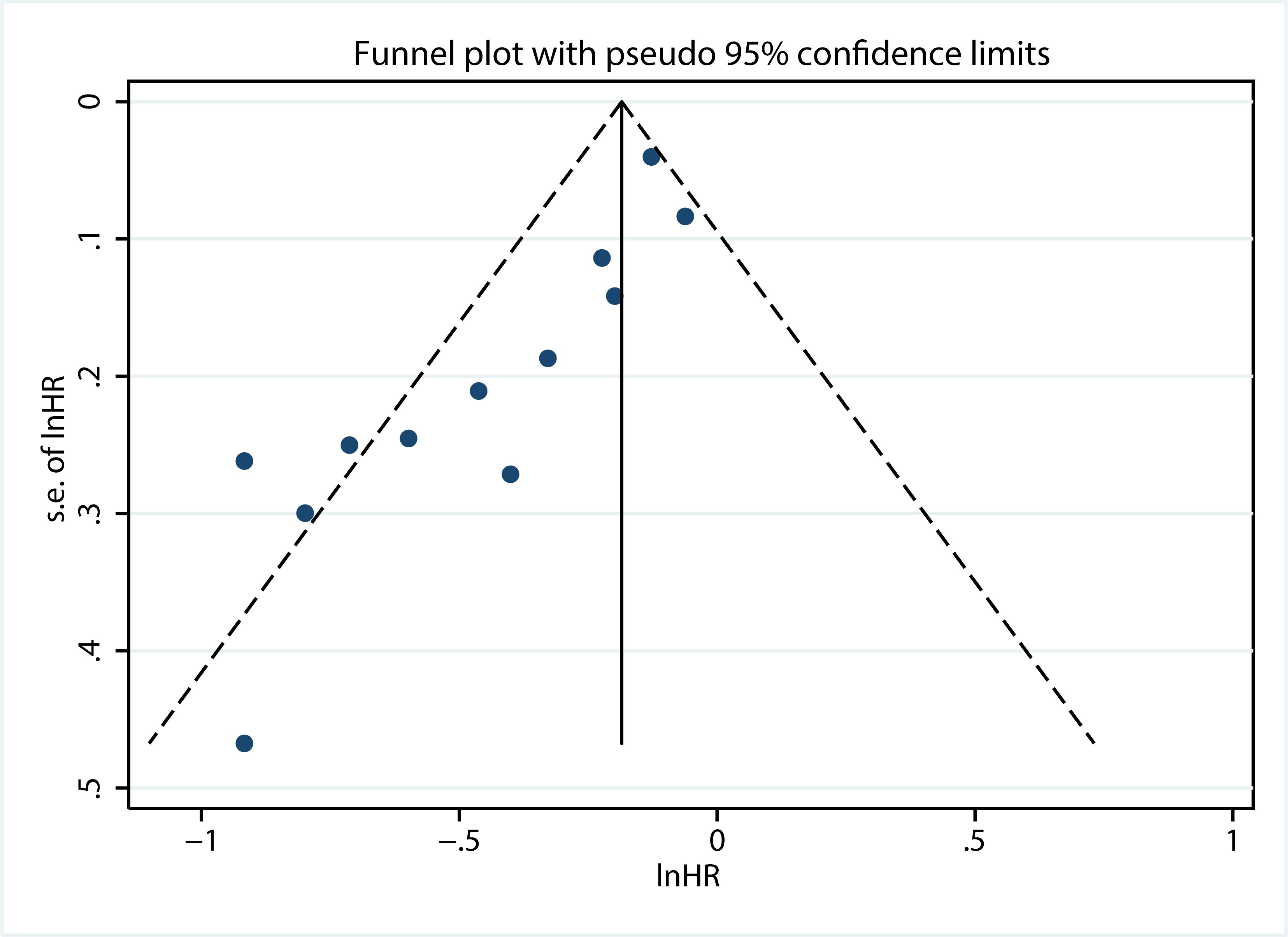

Supplement: Supplementary file 1 [file DataSheet_1.zip › Supplemental materials/SFig 7.tif]

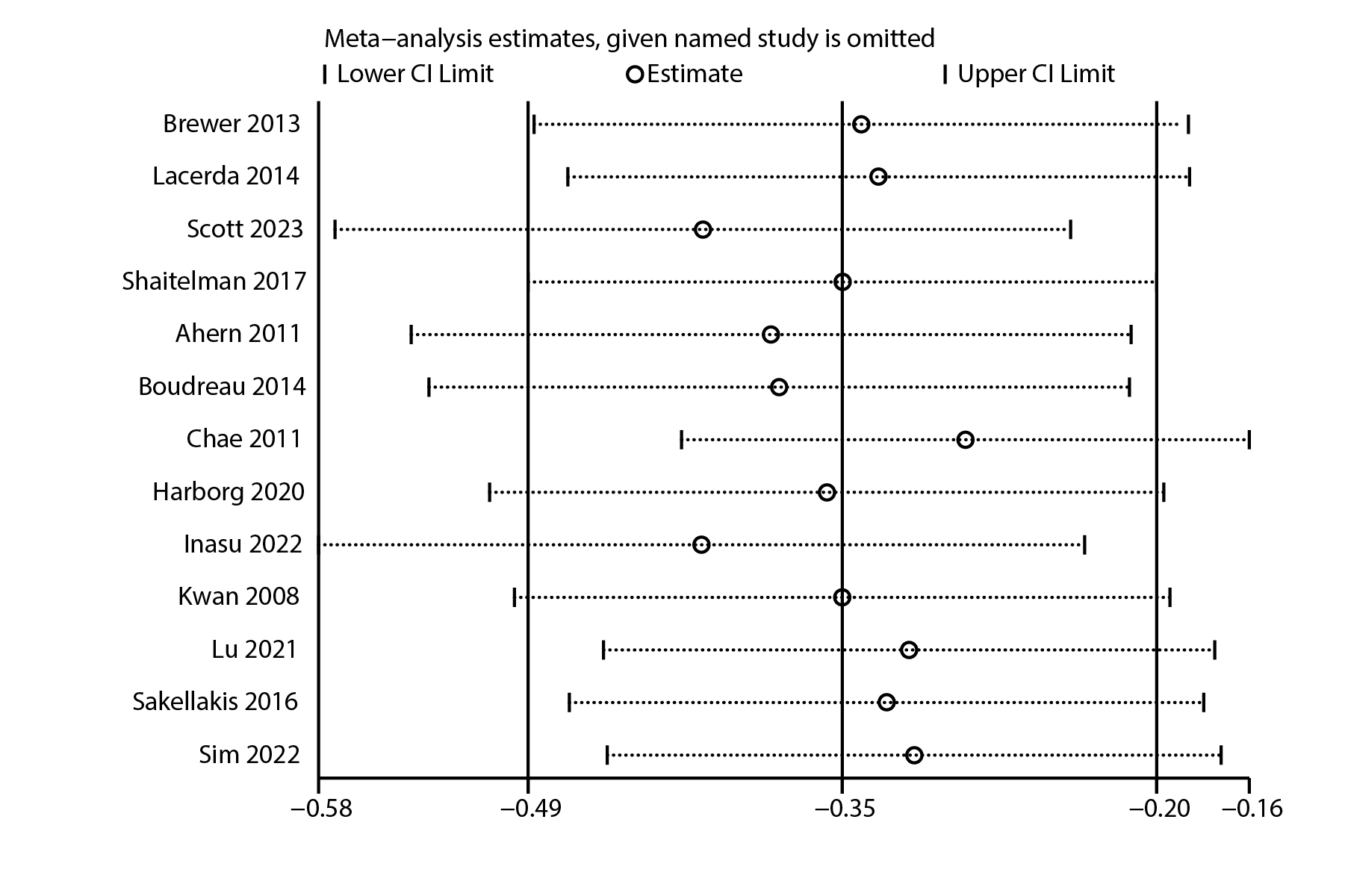

Supplement: Supplementary file 1 [file DataSheet_1.zip › Supplemental materials/SFig 8.tif]

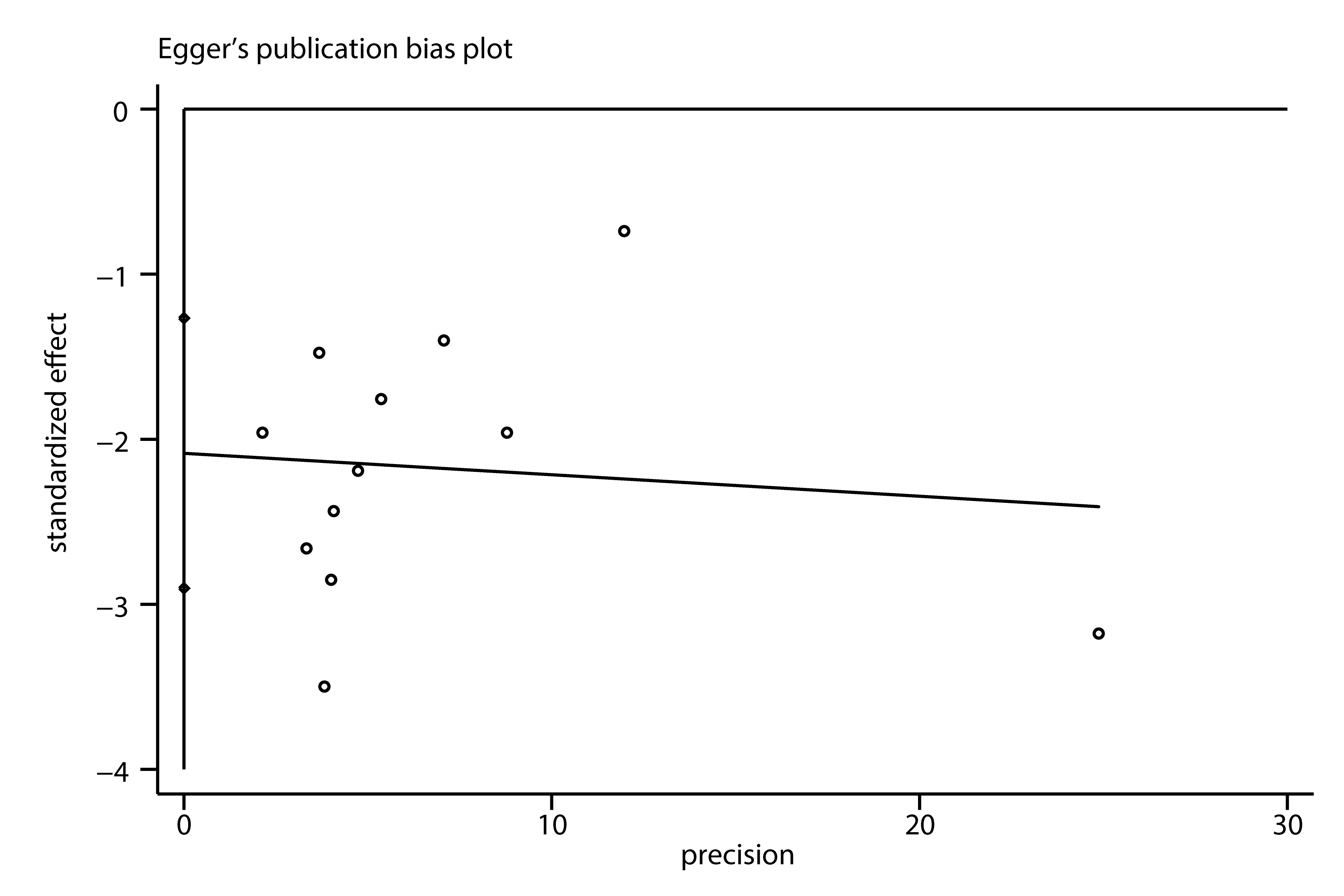

Supplement: Supplementary file 1 [file DataSheet_1.zip › Supplemental materials/SFig 9.tif]
